# Prevalence and Risk Factors for Opioid-Induced Constipation in an Older National Veteran Cohort

**DOI:** 10.1155/2020/5165682

**Published:** 2020-03-29

**Authors:** Fern FitzHenry, Svetlana K. Eden, Jason Denton, Hui Cao, Aize Cao, Ruth Reeves, Guanhua Chen, Glenn Gobbel, Nancy Wells, Michael E. Matheny

**Affiliations:** ^1^Tennessee Valley Healthcare System, Veterans Affairs Medical Center, Nashville, TN, USA; ^2^Vanderbilt University, Nashville, TN, USA; ^3^Vanderbilt University Medical Center, Nashville, TN, USA; ^4^AstraZeneca Pharmaceuticals, Gaithersburg, MD, USA; ^5^University of Wisconsin Madison School of Medicine and Public Health, Madison, WI, USA

## Abstract

**Objectives:**

This research describes the prevalence and covariates associated with opioid-induced constipation (OIC) in an observational cohort study utilizing a national veteran cohort and integrated data from the Center for Medicare and Medicaid Services (CMS).

**Methods:**

A cohort of 152,904 veterans with encounters between 1 January 2008 and 30 November 2010, an exposure to opioids of 30 days or more, and no exposure in the prior year was developed to establish existing conditions and medications at the start of the opioid exposure and determining outcomes through the end of exposure. OIC was identified through additions/changes in laxative prescriptions, all-cause constipation identification through diagnosis, or constipation related procedures in the presence of opioid exposure. The association of time to constipation with opioid use was analyzed using Cox proportional hazard regression adjusted for patient characteristics, concomitant medications, laboratory tests, and comorbidities.

**Results:**

The prevalence of OIC was 12.6%. Twelve positively associated covariates were identified with the largest associations for prior constipation and prevalent laxative (any laxative that continued into the first day of opioid exposure). Among the 17 negatively associated covariates, the largest associations were for erythromycins, androgens/anabolics, and unknown race.

**Conclusions:**

There were several novel covariates found that are seen in the all-cause chronic constipation literature but have not been reported for opioid-induced constipation. Some are modifiable covariates, particularly medication coadministration, which may assist clinicians and researchers in risk stratification efforts when initiating opioid medications. The integration of CMS data supports the robustness of the analysis and may be of interest in the elderly population warranting future examination.

## 1. Introduction

Pain is the most common symptom motivating patients to seek care in the United States [1] and results in significant suffering [2] and expense [3, 4]. Opioids are given to manage pain and are one of the most commonly prescribed drugs in the US with 82.5 prescriptions per 100 persons in 2012 [5]. However, there are a number of potential adverse effects from opioid use, which include physical dependence, nausea, sedation, vomiting, constipation, tolerance, dizziness, respiratory depression, and—less commonly—delayed gastric emptying, immunologic and hormonal dysfunction, muscle rigidity, hyperalgesia, and myoclonus [6].

Among these, constipation is the most common side effect occurring during opioid use. Opioid-induced constipation (OIC) has a frequency of approximately 40% [7–9]. OIC is primarily mediated through the effects of opioids on *μ*-receptors in the gastrointestinal tract, reducing bowel tone, contractility, and luminal fecal moisture [10]. There may also be centrally mediated effects, decreasing autonomic output and reducing gastrointestinal propulsion [10]. In addition, although other side effects of opioid use may diminish over time, constipation likely will not improve [6]. Higher costs are associated with OIC in the nonelderly and elderly patients when compared to patients without OIC—52% to 89% (elderly) higher costs in patients on opioids 90 days or more when compared to patients without OIC [11].

Evidence indicates that veterans are more likely to suffer from chronic pain at a younger age and receive opioids more frequently for this pain than civilians [12–14]. Military service may be associated with experiencing chronic pain earlier in life, whereas for civilians, chronic pain begins to appear in mid-life [12]. In comparative studies, Toblin et al. found that the military had more chronic pain (44.0%) and opioid use (15.1%) [13] than the civilian group (26.0% and 4.0%) [14]. Among male veterans enrolled in primary care, 50% reported pain [15]. Approximately 50% of veterans with chronic noncancer pain (1.44 million) were receiving an opioid according to a 2014 study [16]. Despite the use of opioids for chronic noncancer pain, there are little data on the prevalence and variables associated with OIC in this population.

Understanding the prevalence and associated variables that increase the probability of OIC would facilitate recommending treatment directed to the patients most likely to benefit. Higher-risk patients might be given more specific therapy if standard therapy is less effective [17].

The purpose of this study was to evaluate the prevalence and covariates associated with opioid-induced constipation (OIC) as evidenced by administrative coding or prescriptions/fills of new/changed laxatives in patients receiving care from the national Veterans Health Administration (VA). The findings will help raise awareness of OIC and may assist clinicians in risk identification when initiating opioid medications.

## 2. Materials and Methods

### 2.1. Study Setting

The research was designed as a retrospective observational cohort study of the national VA patient population of patients aged ≥65 years at the time of initiation of opioids from 1 January 2008 to 30 November 2010 with integrated data from the Center for Medicare and Medicaid Services (CMS). When veterans (and all United States [US] citizens) reach age 65, they become eligible for a free form of national health insurance administered by CMS, Medicare with some deductibles and copayments. The free coverage component available from Medicare (inpatient, skilled care, hospice, and limited home health) may be appealing to some veterans and was available from CMS for research. CMS data was only included if the individual was also a VA patient. Veterans who never received care at a VA facility were not included. The outcome of interest was opioid-induced constipation (OIC) following initiation of opioids. The exposure of interest was sustained exposure to newly initiated opioids prescribed through a VA inpatient/outpatient pharmacy or a CMS prescription (outpatient). Evaluation of a large set of candidate clinical factors, including patient demographics, administrative codes, medications, laboratory results, and healthcare utilization, was executed to assess their relationships to the diagnosis or treatment (prescription/procedure) of constipation in the presence of opioid use (documented in the electronic health record (EHR)). All elements of the study design are described in detail below. The study protocol was approved by the VA Tennessee Valley Healthcare System Institutional Review Board.

### 2.2. Exposure Data Sources

The data included International Classification of Diseases, Ninth Revision, Clinical Modification (ICD-9-CM) [18] diagnosis/procedure codes, Common Procedural Terminology (CPT) [19] procedure codes, medication records, encounters, demographics, and laboratory results (VA only). In the United States, ICD-9-CM use started with the World Health Organization's Ninth Revision, International Classification of Diseases (ICD-9), but additions and modifications have been made by the National Center for Health Statistics (NCHS) and the CMS. The American Medical Association (AMA) developed the CPT coding system. Both CMS and VA provided data. VA data was extracted from the VA Electronic Health Record, Computerized Patient Record System (CPRS), stored within the Corporate Data Warehouse (CDW) within the VA Informatics and Computing Infrastructure [20–22]. Further detail on data fields was available at http://vaww.vinci.med.va.gov/vincicentral/default.aspx.

The data availability window was from 1 January 2008 to 30 November 2010, allowing for an additional 24 months of baseline data collections and 1 month of outcome ascertainment for all patients.

The CMS institutional and physician supplier Medicare/Medicaid claims were obtained through the VIReC HSR&D service center. Data were linked between the VA and Medicare/Medicaid sources through patient social security number as previously published by VIReC [23–25]. The CMS Medicare/Medicaid data included diagnostic codes and procedures (ICD-9-CM (18) and CPT (19)) as well as outpatient medications under Medicare Part D coverage. Medicare Part D is an optional drug benefit in the United States covering outpatient prescriptions via beneficiary paid insurance plans.

### 2.3. Definition

The key exposure was a sustained exposure to opioids, defined as ≥30 continuous days use of any opioid medication (Supplementary [Supplementary-material supplementary-material-1]). Both inpatient (bar-coded medication administration, BCMA) and outpatient fill records were utilized in this assessment. We calculated medication exposure windows with an algorithm to address stockpiling and short-term fill gaps not likely to represent a gap in therapy, implemented approximating a previously validated algorithm [26]. In brief, we defined continuous fill records directly from bar-coded medication administrations when patients were in the hospital (BCMA-VA only), and through the issue date and number of fill days allowing for a fourteen-day gap in therapy when patients were not hospitalized. Gaps beyond this limit were broken into separate exposure windows for purposes of analysis, and opioid dose volume was not considered in the exposure ascertainment.

### 2.4. Outcome Definition

The primary outcome variable was opioid-induced constipation (OIC), defined as all-cause constipation occurring in the time period immediately following opioid initiation. Because there was no specific administrative code for OIC, we utilized an aggregate definition for a constipation event that included the following: (1) ≥1 medical claim with an ICD-9 diagnosis code for constipation (Supplementary [Supplementary-material supplementary-material-1]) during the opioid era, OR (2) constipation defined by a new initiation or a change in laxative during the opioid era (Supplementary [Supplementary-material supplementary-material-1]) and excluding any new laxative initiations occurring in the first 3 days of the start of the opioid (the new laxative initiations/orders at the start of the opioid would instead be classified as preventative laxatives), OR (3) a constipation related procedure code during the opioid era (Supplementary [Supplementary-material supplementary-material-1]). The definition of constipation for retrospective data was drawn from industry experts and prior research [27]. Patients were only eligible for OIC during the period in which they were exposed to opioids, as defined by the exposure window.

This retrospective definition of OIC differs from the recently added Rome IV criteria for OIC. The OIC criteria published in 2016 requires the presence of an opioid (new, changed, or increasing) with new or worsening symptoms of constipation. Specifically, at least two of the following constipation symptoms must occur with at least 25% of defecations: (a) straining, (b) lumpy or hard stools, (c) sensation of incomplete evacuation, (d) sensation of anorectal obstruction/blockage, (e) manual maneuvers to facilitate, and (f) fewer than three spontaneous bowel movements a week. In addition, loose stool would be rare without laxatives [28].

### 2.5. Cohort Inclusion/Exclusion Criteria

The initial patient inclusion criteria were all patients aged ≥65 years who newly initiated sustained opioid therapy between 1 January 2008 and 30 November 2010 and subsequently ended use. Full exposure windows were required to define ascertainment windows for the outcome of interest. We further restricted the cohort to patients who were obtaining regular care from the VA, defined ≥1 inpatient or outpatient encounter per year at a VA facility in the 24 months prior to the index date. To ensure that patients were opioid naïve, patients with any record of an opioid exposure in the 12 months prior to the index date were excluded. To ensure the patients were being treated for noncancer pain, patients were excluded if they had ≥2 administrative claims for a cancer diagnosis (excluding ICD-9 codes (140.xx–209.3x, 230.xx–234.xx) during the 12 month pre-index period (Supplementary [Supplementary-material supplementary-material-1]). Some patients had multiple opioid eras in the study period. In those cases, we only examined the first opioid era.

### 2.6. Covariates/Risk Factors

We included a wide array of candidate risk factors for the outcome of interest, both to assess associations and to adjust for other factors that could potentially cause constipation. We included 5 demographic variables, 23 conditions based on administrative codes, 20 laboratory test results, and 37 VA class medication exposures and other medication variables. Risk factors were assessed for the year prior to the beginning of the opioid exposure. All details of the definitions of included covariates are presented in Supplementary [Supplementary-material supplementary-material-1].

Administrative code-based conditions were defined using ICD-9-CM. Except for age and laboratory results, covariates were converted to binary yes/no variables. From all the administrative candidate variables, almost all variables that were present in <1% of the cohort variables were removed from consideration, except for rare race values, gastrointestinal (GI) anatomical trauma, hyperkalemia, and irritable bowel syndrome (full list, Supplementary [Supplementary-material supplementary-material-1]). In addition, because of low frequency, a group of possible sequelae to constipation were combined to a single variable: GI anatomical trauma. This variable included gastrointestinal perforation, bowel perforation, rectal prolapse, rectocele, megacolon, impaction, and anal fissures fistula.

A list of the included laboratory results is presented in Supplementary [Supplementary-material supplementary-material-1]. Laboratory test variables that were missing in 60% or more of the cohort were excluded. For those retained, an average value was calculated for the year prior to the opioid start date. The laboratory tests considered but excluded are listed in Supplementary [Supplementary-material supplementary-material-1].

Pre-index medication variables were determined as exposed/unexposed, indicating if a patient took the medication or not during the 12 months prior to the index date. Medication exposure covariates were aggregated at the level of VA Drug Classes for the year prior to the opioid initiation date. Because of low frequency values, the rectal laxative drug class was combined with the general laxative drug class under the name “Laxatives, any route.” The VA Drug Classes controlled vocabulary mappings are publicly available within the National Library of Medicine's Unified Medical Language System [29] and have crosswalks to NDC [30], RxNorm [31], ATC [32], and VA Product codes [33] for external reproducibility. To facilitate a comparison of VA drugs used to define laxatives included in ATC drug classes, Supplementary [Supplementary-material supplementary-material-1] includes a comparison by drug name.

Constipating medications were handled as a special class, developed using the definition of anticholinergic drugs identified by Boustani and colleagues, and supplemented by a group of pharmacy industry experts from those included in prior studies [34]. If a drug ingredient was included in the constipating medication variable, it was removed from the standard VA drug class (see Supplementary Materials). In this way, the confounding effect was minimized, so the same drug was not repeated in multiple covariates. In addition, the supplemental drug list was investigated to confirm that constipation was listed as a side effect either by drug or by drugs within the drug class. If constipation was confirmed as a side effect in the drug's warnings in Lexicomp, [35] the drug was added to the constipating medication drugs. A full list of all medications aggregated into the constipating medications is available in Supplementary [Supplementary-material supplementary-material-1].

Because of the need to carefully evaluate constipating medications in the context of opioid use and the anticipation that constipating medications close to the start of the opioid era would be the most likely to influence the outcome, the temporal relationship to exposure of these medications and opioid use was explored through creation of variables with three timespans: (1) Prior Use Constipating Medications 1 Year To 61 Days, (2) Prior Use Constipating Medications 60 to 31 Days, and (3) Prior Use Constipating Medications 30 to 1 Day. The timespans were mutually exclusive, so an era could belong to only one of the timespans.

Lastly, several other exposure and outcome related variables were generated, including prevalent laxative (any laxative started prior to the opioid exposure with a prescription amount that continued into at least the first day of opioid exposure) and preventative laxative (any laxative that began from the first to the third day of opioid exposure). For descriptive purposes, prior constipation found in diagnosis codes, in procedure codes, and in laxative medications additions or changes was captured.

### 2.7. Missing Data

Management of missing data is important for any secondary use retrospective cohort study [36]. For a subject to be included in a regression model, each occurrence of each risk variable for this subject should not be missing. Except for laboratory test data, clinical information was assumed to be negative or not present when it was not found in the electronic health record data. This is a common assumption in administrative data, and for many of the variables, point of care data collection provides additional assurances that the lack of mention is a valid absence of that condition or medication.

Missing values among laboratory tests were addressed in a different fashion because they were discretely present or missing in addition to being normal, low, or high when present. All these states were captured in the electronic health record, and each candidate variable defined with laboratory data was assessed for the proportion of missing values. Missing values in tests were imputed using multiple imputation with predictive mean matching [37]. This method is superior to single imputation and has been shown to be robust for high proportions of missing values [36]. The variables used to impute missing values for the laboratory test values included both patient characteristics listed in Supplementary [Supplementary-material supplementary-material-1] and nonmissing values of the same laboratory tests listed in Supplementary [Supplementary-material supplementary-material-1].

### 2.8. Statistical Analysis

Time to constipation was summarized using a Kaplan–Meier plot. The adjusted association of time to constipation with opioid use was analyzed using Cox proportional hazard regression. Our final model was adjusted for the 85 included variables. Hazard ratios (HR) with 95% confidence intervals were reported for each variable.

Date of the first fill or administration for an opioid medication was marked as the index date. The discrimination ability of the models was assessed using the C-index [38]. Variables were considered statistically significant if the two-sided *p* value was 0.05 or lower. Because the sample size was large and likely to produce significant results that might not be clinically significant, we also decided to only discuss hazard ratio results outside the range 0.9 to 1.1 where the confidence intervals did not cross 1. The analysis was completed using statistical language R Version 3.3.2 [39]. The R statistical computing and graphics software is available free as an open source package.

## 3. Results

**Figure 1:**
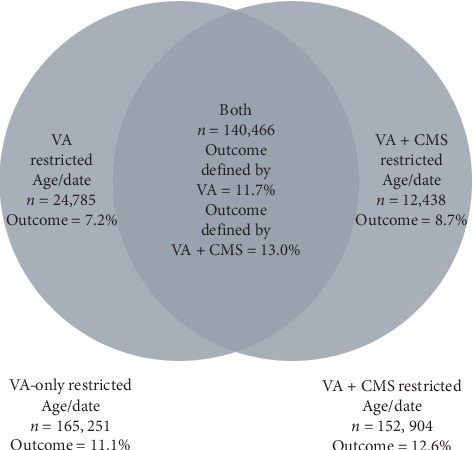
Venn diagram of differences in the cohort size and ascertainment. When data from the Center for Medicare and Medicaid Services (CMS) are added to Veterans Health Administration (VA) electronic medical record, patients are added to and subtracted from the cohort.

After obtaining the VA data we linked the VA data to the CMS data creating a merged dataset. The addition of the CMS data both added and subtracted patients to and from the cohort. To demonstrate how the CMS data changed the cohort, we compared the VA + CMS cohort to a sample that matched the study period and age restrictions of the VA + CMS cohort but did not include the CMS data. In the Venn diagram in Figure 1 the differences in the cohort size and ascertainment are recorded. The addition of the CMS data allowed 12,438 additional patients to meet the inclusion criteria. On the other hand, the CMS data required that 24,785 patients be dropped from the sample because, with the additional data supplementation, the patients met exclusion criteria. The prevalence rate for the 152,904 veterans in the VA + CMS cohort was 12.6%.

Demographic characteristics for the cohort are described in Table 1. Of the 152,904 veterans in the cohort, 98% were male and 74% were white. The mean age for the cohort was 76 years [SD 7.35].

Clinical characteristics of the cohort are described in Table 2. Hypertension occurred most frequently in 83% of the cohort. The next most frequent diagnoses were coronary artery disease (42%), diabetes mellitus (40%), and osteoarthritis (37%).

The outcome of constipation was identified through medication (12%), diagnosis (4%), and procedure codes (0.03%). In addition, 87% of patients had previous constipating medication use within 30 days, 2% had constipating medication use from 31 to 60 days, and 6% of patients had constipating medication use from 61 days to 1 year before the index date of opioid therapy. A prior history of constipation was present for 9% of the cohort by diagnosis and 0.03% by procedure codes. In addition, 18% would have had a prior history of constipation based on laxative use.

The median duration for an opioid era for the cohort was 30 days (interquartile range: 30–51) with a maximum of 1024 days (2.88 years). A relatively low proportion of the cohort received a laxative prescription (over-the-counter included) along with the opioid prescription (5%).

**Figure 2:**
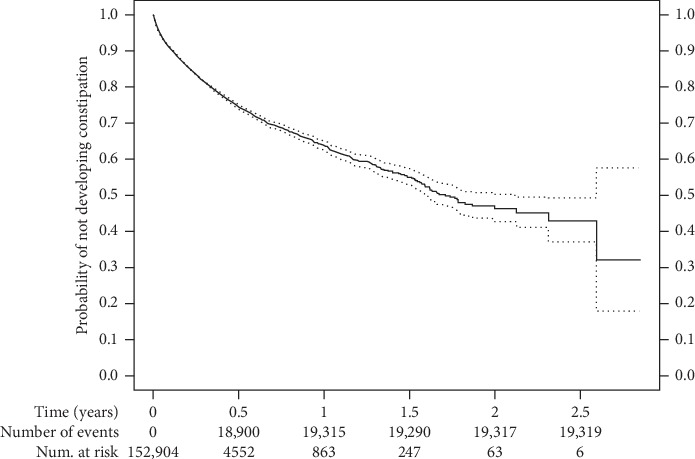
Kaplan–Meier diagram of the relationship between probability of not developing (provider documenting/treating) constipation and the duration of opioid use.

The Kaplan–Meier for the cohort (Figure 2) shows the relationship between probability of not developing (provider documenting/treating) constipation and the duration of opioid use. Patients in the cohort had a nearly 30% probability of developing (provider documenting/treating) constipation after they took an opioid for about half a year. The chance increased to nearly 35% after they took an opioid for about 1 year.

**Figure 3:**
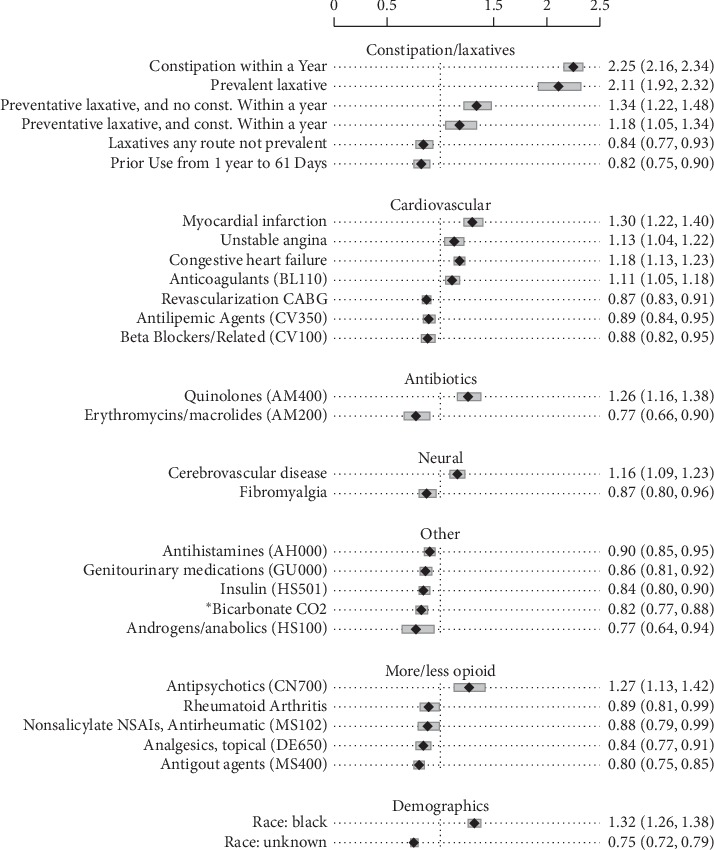
Regression on the adjusted association of variables to time of constipation with opioid use. Variables with effect size outside 0.9 to 1.1 range and variables with confidence intervals that crossed 1 were excluded (*n* = 152,904).

The Cox Regression C-index [39] (indicating greater concordance between actual and predicted time to constipation) was 0.672 (95% CI [0.667–0.676]) in the cohort. The analysis included 85 variables, of which, 29 reached significance/effect size inclusion criteria. Variables with effect size outside 0.9 to 1.1 range and variables with confidence intervals that crossed 1 were excluded (see Table 3). The variables included in the regression shown in Table 3 and Figure 3 are grouped in categories by demographics, condition, laboratory values, and/or treatment.

For the constipation/laxative variables, HR ranged from 1.18 to 2.25 within the cohort. Constipation within a year had the largest HR positively associated with a higher probability of constipation. Among the laxative variables, only laxatives any route not prevalent, i.e., laxatives taken in the prior year that did not extend into the opioid era index date, was negatively associated (HR = 0.84, 95% CI [0.77, 0.93] in the cohort). Preventative laxatives had a positive association with the rate of occurrence of the study-defined constipation outcomes (see Supplementary Tables [Supplementary-material supplementary-material-1]–[Supplementary-material supplementary-material-1]) both with and without a history of constipation, HR = 1.18 (95% CI [1.05, 1.34]) and 1.34 (95% CI [1.22, 1.48]), respectively.

Constipating medications that were stopped >61 days prior to the opioid era index date were significantly negatively associated with probability of provider documenting/treating OIC, HR = 0.84, 95% (CI [0.75, 0.90]). Constipating medication use that continued to within 30 days of the opioid index date was common, including 86.53% of the subjects.

Among the demographic variables, African American race was positively associated with probability of provider documenting/treating constipation, HR = 1.32 (95% CI [1.26, 1.38]).

The cardiovascular variables myocardial infarction, unstable angina, congestive heart failure, and anticoagulants (BL110) had positive associations with probability of constipation, with HR ranging from 1.11 to 1.3. However, negative associations appeared for three variables associated with treatment of cardiovascular disease—antilipemic agents, beta blockers, and revascularization CABG—with HR ranging from 0.87 to 0.89.

Several medications used in the treatment of pain—non-salicylate NSAIs and antirheumatic (MS102), analgesics, topical (DE650), and antigout agents (MS400)—were negatively associated with the probability of OIC (with HR ranging from 0.80 to 0.89). Treatment with insulin was negatively associated with probability of OIC (HR = 0.84, 95% CI [0.80, 0.90]).

Antipsychotics were positively associated with probability of OIC (HR = 1.27, 95% CI [1.13, 1.42]).

## 4. Discussion

This study evaluated prevalence and covariates associated with the likelihood of developing OIC as evidenced by administrative coding or prescriptions/fills of new/changed laxatives within a large national veteran cohort. The study found that, after six months on opioids, there was a higher probability of provider documenting/treating OIC. The novel findings of this study included 12 significant covariates positively associated with probability of constipation in the setting of opioid use and thereby likely for OIC. The variables that showed the largest associations with OIC were in the category constipation/laxatives, including constipation within a year and prevalent laxative. Among the 17 negatively associated covariates, the largest were erythromycins, androgens/anabolics, and unknown race.

The prevalence of OIC in this sample was lower than what others have reported [7–9]. For example, a meta-analysis reported that OIC occurred in up to 41% of patients [8, 40]. In a survey, 40.3% of opioid users reported constipation compared to 7.6% of controls. However, the survey used targeted questions to ascertain constipation. This study used medication fills and administrative codes collected from the electronic health record (EHR) linked to CMS Medicare claims data. Irvine et al. [41] found that only 28.9% of their study patients went to their provider for constipation (thus eligible to appear in the EHR). One survey study specifically asked why patients experiencing constipation did not discuss it with their provider. Only 4% of patients said it was because constipation was not a problem [42]. Among the reasons why patients did not discuss their constipation with a provider were previous discussions, embarrassment, and concern about changing the pain medication. The study also found that 59% of patients who did not discuss their OIC with their provider reported that it was because they had already brought up their OIC on previous visits [42].

The Kaplan–Meier curve indicates that the risk of developing OIC is higher after 6 months of continuous use of opioids. Although it is reasonable to expect increased risk over time, to our knowledge no data have been published on increased risk by duration of opioid use. It is possible that the risk of developing OIC increased as patients came back into contact with the healthcare system and were documented to have OIC, and it is also possible that additional exposures of acute illness and other medications promoted increasing rates of OIC over time. In addition, it may be that patients are well controlled by initially addressing opioid-induced constipation on their own with changes in diet or over-the-counter products. It is the patients for whom OIC interferes with daily activities and who become dissatisfied over time that need to be recognized.

The results indicate that, despite national VA practice recommendations, a low proportion of patients, only 5%, were prescribed a preventative laxative treatment with the opioid. This low rate of laxative prophylaxis is similar to a national sample of patients discharged from an emergency department [43] and a small sample of ambulatory cancer patients with opioid prescriptions [44]. The lack of an association between preventative laxatives and the study-defined constipation outcome could be confounded by increases in ascertainment, reporting, and documentation of OIC among those taking preventative laxatives, although one would expect that opioid users would be frequently queried by their healthcare provider about any ongoing all-cause constipation.

A potentially novel finding was the association of probability of OIC with cardiovascular disease and treatment. To our knowledge, there was only a single prior study that found a similar association in a cohort of 93,676 women [45]. There was also a study that found that patients on chronic opioids were at increased risk for adverse cardiovascular outcomes [46].

There are several conditions found to be associated with OIC with prior supporting evidence for their association with constipation, although most are reported in all-cause chronic constipation, which is mechanistically distinct from OIC. In the context of this study, patients with covariates for all-cause chronic constipation may be more susceptible to OIC by interactions between mechanisms. Diabetes, quinolones, erythromycin, and antigout medications have been associated with all-cause chronic constipation [47–54]. However, it is important to note that this work extends the literature by reporting these covariates among a cohort experiencing constipation due to incident opioid exposure. The increased risk of OIC with quinolones may occur because of an interaction between mechanisms of action.

On the other hand, erythromycin had a negative association. This is consistent with prior literature in which erythromycin has been shown to reduce colonic transit time; however, the mechanism of action is not well understood [50, 51]. One antigout medication, colchicine, has been researched for possible use in all-cause chronic constipation [48].

More directly related to OIC, elevated bicarbonate was negatively associated with OIC. Intestinal excretion of bicarbonate may modulate fecal luminal moisture [55].

### 4.1. Limitations

This study has some limitations. First, the veteran population studied has a higher percentage of males and is older than the general population [56]. Second, when compared to civilians, veterans tend to be disproportionately African American, male, less educated, more likely out of the workforce, of lower income, and have more comorbidities [56]. Over-the-counter laxatives may not always be reported to the providers and would not then be included in the health record data used for this study. Third, the study used secondary data which did not include a systematic measure of the severity of constipation, quality of life, or components of the Rome IV OIC criteria. All of the relationships found in this study were associations and do not provide statistical evidence of causal conclusions [57]. Fourth, although the population was enriched through a continuity of care inclusion criteria, veterans may receive care outside of the VA and thus be missing data in this analysis. The addition of CMS data was an attempt to ameliorate this impact by analyzing the elderly population in which ascertainment was more complete.

## 5. Conclusion

This study adds to the literature by analyzing a large national cohort of incident opioid users in a setting where all or most of the clinical care is received within the VA/Medicare. There were several novel covariates found that are also found in the all-cause chronic constipation literature but have not been reported for opioid-induced constipation. Some of these are modifiable variables, particularly medication coadministration, which may assist clinicians and researchers in risk stratification efforts when initiating opioid medications. Lastly, integration of the CMS data supports the robustness of the analysis and may be of interest in the elderly population warranting future examination.

## Figures and Tables

**Table 1:** Demographics of veterans covered by the study.

	VA and CMS patients count (percent/SD)	
Characteristic				
Total	152,904	(100.00)
Male	149,092	(97.51)
Female	3,812	(2.49)
Age (mean [SD])	76	(7.35)

*Race*		
White	112,517	(73.59)
African American	14,672	(9.60)
Unknown	22,785	(14.90)
Asian/Hawaiian/Pacific Islander	2,130	(1.39)
American Indian, Alaskan Native	800	(0.52)

*Note.* VA: Veterans Health Administration; CMS: Center for Medicare and Medicaid Services.

**Table 2:** Clinical characteristics of veterans covered by the study.

Variable	VA + CMS count/percent	
*Outcomes*		
Constipation identification through diagnosis	6,872	4.49%
Constipation identification through procedure	43	0.03%
Constipation identification through medication	17,810	11.65%

*Medications*		
Laxative use prior year	28,143	18.41%
Constipating Meds-Prior Use from 1 year to 61 days	9,076	5.94%
Constipating Meds-Prior Use from 60 to 31 days	3,431	2.24%
Constipating Meds-Prior Use from 30 to 1 day	132,303	86.53%
Laxative use prior to opioid era	32,593	21.32%
Prevalent laxative	9,446	6.18%
Preventative laxative	7,514	4.91%

*Medical conditions*		
Prior constipation diagnosis	13,071	8.55%
Prior constipation procedure	44	0.03%
Impaction	Less than 11	0.00%
Hemorrhoids	15,160	9.91%
Gastrointestinal anatomical trauma	556	0.36%
Cerebrovascular disease	19,253	12.59%
Chronic kidney disease	23,051	15.08%
Colitis	1,033	0.68%
Congestive heart failure	25,960	16.98%
Coronary artery disease	63,509	41.54%
Diabetes mellitus	60,628	39.65%
Diabetic neuropathy	5,639	3.69%
Fibromyalgia	4,030	2.64%
Gastroparesis	899	0.59%
Hypercalcemia	750	0.49%
Hyperkalemia	4,838	3.16%
Hyperparathyroidism	852	0.56%
Hypertension	126,939	83.02%
Irritable bowel syndrome	1,236	0.81%
Megacolon	57	0.04%
Migraine headache	6,637	4.34%
Multiple sclerosis	348	0.23%
Myocardial infarction	5,103	3.34%
Myopathies	7,396	4.84%
Osteoarthritis	56,514	36.96%
Parkinson's disease	3,519	2.30%
Revascularization CABG	18,570	12.14%
Rheumatoid arthritis	3,166	2.07%
Stable angina	13,540	8.86%
Stroke	11,193	7.32%
Unstable angina	17,952	11.74%

*Note.* VA: Veterans Health Administration; CMS: Center for Medicare and Medicaid Services; CABG: coronary artery bypass grafting; “Constipating Meds-Prior Use” refers to the use of medications that have constipation as a side effect such as iron supplements.

**Table 3:** Hazard ratios of significant variables in veterans covered by the study.

Variable	Effect	Significance	Lower confidence interval	Upper confidence interval
Constipation/laxatives				
Constipation within a year	2.25	<0.0001	2.16	2.34
Prevalent laxative	2.11	<0.0001	1.92	2.32
Preventative laxative, and no const. within a year	1.34	<0.0001	1.22	1.48
Preventative laxative, and const. within a year	1.18	0.007	1.05	1.34
Laxatives any route not prevalent	0.84	<0.001	0.77	0.93
Prior use (constipating medications) from 1 year to 61 days	0.82	<0.0001	0.75	0.90
Cardiovascular				
Myocardial infarction	1.3	<0.0001	1.22	1.40
Unstable angina	1.13	0.003	1.04	1.22
Congestive heart failure	1.18	<0.0001	1.13	1.23
Anticoagulants (BL110)	1.11	<0.001	1.05	1.18
Antilipemic agents (CV350)	0.89	<0.001	0.84	0.95
Beta blockers/related (CV100)	0.88	<0.001	0.82	0.95
Revascularization CABG	0.87	<0.0001	0.83	0.91
Antibiotics				
Quinolones (AM400)	1.26	<0.0001	1.16	1.38
Erythromycins/macrolides (AM200)	0.77	<0.001	0.66	0.90
Neural				
Cerebrovascular disease	1.16	<0.0001	1.09	1.23
Fibromyalgia	0.87	0.005	0.80	0.96
Other				
Antihistamines (AH000)	0.9	<0.001	0.85	0.95
Genitourinary medications (GU000)	0.86	<0.0001	0.81	0.92
Insulin (HS501)	0.84	<0.0001	0.80	0.90
^*∗*^Bicarbonate CO_2_	0.82	<0.0001	0.77	0.88
Androgens/anabolics (HS100)	0.77	0.009	0.64	0.94
More/less opioid				
Antipsychotics (CN700)	1.27	<0.0001	1.13	1.42
Rheumatoid arthritis	0.89	0.03	0.81	0.99
Nonsalicylate NSAIs, antirheumatic (MS102)	0.88	0.031	0.79	0.99
Analgesics, topical (DE650)	0.84	<0.0001	0.77	0.91
Antigout agents (MS400)	0.8	<0.0001	0.75	0.85
Demographics				
Race African American	1.32	<0.0001	1.26	1.38
Race unknown	0.75	<0.0001	0.72	0.79

*Note.* Drug classes are based on the Veterans Health Administration formulary. The five-character acronym following the drug class is the abbreviation for the drug class in the Veterans Health Administration. CABG: coronary artery bypass grafting.

## Data Availability

The variable definition data used to support the findings of this study are included within the supplementary information files. The patient data used to support the findings of this study are restricted by the Institutional Review Board and VA-wide security standards for data security requiring that the database be secured within the VA's firewall and maintained by the Office of Information and Technology (OI&T), in order to protect patient privacy. Data are available from Michael E. Matheny (Micheal.Matheny@va.gov) for researchers who meet the criteria for access to confidential data.

## Supplementary Materials

Supplementary Materials This section includes the following: opioid medications included (Table 1); codes used to identify constipation, e.g., diagnosis (Table 2) and procedures (Table 4); laxative medications included (Table 3); codes for cancer inclusion subset from ICD-9-CM 140-239 Neoplasms (Table 5); definition of covariates and risk factors (Table 6); laboratory tests excluded (Table 7); medications defined as constipating medications (Table 8); patient characteristics used for imputation of missing data (Table 9); and laboratory tests used for imputation of missing data (Table 10).
