# Supplementary material for: Prevalence and Risk Factors for Opioid-Induced Constipation in an Older National Veteran Cohort
Source: Pain Res Manag. 2020 Mar 29;2020:5165682. doi: 10.1155/2020/5165682 (PMC7149448; doi:10.1155/2020/5165682)
Supplement: Supplementary Materials — This section includes the following: opioid medications included (Table 1); codes used to identify constipation, e.g., diagnosis (Table 2) and procedures (Table 4); laxative medications included (Table 3); codes for cancer inclusion subset from ICD-9-CM 140-239 Neoplasms (Table 5); definition of covariates and risk factors (Table 6); laboratory tests excluded (Table 7); medications defined as constipating medications (Table 8); patient characteristics used for imputation of missing data (Table 9); and laboratory tests used for imputation of missing data (Table 10). [file 5165682.f1.docx]

Appendix 1

Table 1: Opioid Medications

| **Opioid Medication** |
| --- |
| Morphine |
| Oxycodone |
| Hydromorphone |
| Alfentanil |
| Buprenorphine |
| Fentanyl |
| Meperidine |
| Remifentanil |
| Sufentanil |
| Hydrocodone |
| Codeine |
| Methadone |
| Propoxyphene |
| Pentazocine |
| Butorphanol |
| Levorphanol |
| Nalbuphine |
| Oxymorphine/Oxymorphone |
| Opium |
| Tramadol |
| Dihydrocodeine |

Table 2: ICD-9 Codes Used to Identify Constipation

| **Disease Diagnosis** | **ICD-9 dx code** |
| --- | --- |
| Changes in bowel habits | 787.99 |
| Constipation | 564.0x |
| Fecal impaction | 560.32 |
| Impaction of intestine unspecified, other | 560.30, 560.39 |
| Large bowel obstruction: Acute pseudo-obstruction of intestine | 560.89 |
| Megacolon (not congenital) | 564.7x |
| Unspecified intestinal obstruction | 560.9x |

Table 3: Laxatives used to identify Constipation Outcome Laxative Use Mapped by Drug Name to ATC Drugs

| Drug class description | Drug name | ATC Category | ATC Drug Code and Name |
| --- | --- | --- | --- |
| RS300 Laxatives,rectal | Benzocaine/docusate |  |  |
| GA204 Stimulant laxatives | Bisacodyl | A06AB Contact laxatives | A06AB02 Bisacodyl |
| GA202 Hyper-osmotic laxatives | Bisacodyl |  |  |
| GA200 Laxatives | Bisacodyl |  |  |
| GA209 Laxatives,other | Bisacodyl |  |  |
| RS300 Laxatives,rectal | Bisacodyl | A06AG Enemas | A06AG02 Bisacodyl |
| GA200 Laxatives | Bisacodyl tannex | A06AB Contact laxatives | A06AB52 Bisacodyl, combinations |
| GA209 Laxatives,other | Bisacodyl/electrolytes/peg-3350 | A06AB Contact laxatives | A06AB52 Bisacodyl, combinations |
| GA200 Laxatives | Bisacodyl/magnesium citrate | A06AB Contact laxatives | A06AB52 Bisacodyl, combinations |
| GA209 Laxatives,other | Bisacodyl/magnesium citrate | A06AB Contact laxatives | A06AB52 Bisacodyl, combinations |
| GA200 Laxatives | Bisacodyl/magnesium citrate/sodium phosphate dibasic and sodium phosphate monobasic | A06AB Contact laxatives | A06AB52 Bisacodyl, combinations |
| GA200 Laxatives | Bisacodyl/sodium phosphate dibasic and sodium phosphate monobasic | A06AB Contact laxatives | A06AB52 Bisacodyl, combinations |
| RS300 Laxatives,rectal | Bisacodyl/sodium phosphate dibasic and sodium phosphate monobasic | A06AB Contact laxatives | A06AB52 Bisacodyl, combinations |
| GA204 Stimulant laxatives | Bryonia |  |  |
| GA201 Bulk-forming laxatives | Calcium polycarbophil | A06AC Bulk-forming laxatives | A06AC08 polycarbophil calcium |
| GA200 Laxatives | Calcium polycarbophil | A06AC Bulk-forming laxatives | A06AC08 polycarbophil calcium |
| GA204 Stimulant laxatives | Casanthranol |  |  |
| GA200 Laxatives | Casanthranol |  |  |
| GA200 Laxatives | Casanthranol/docusate |  |  |
| GA209 Laxatives,other | Casanthranol/docusate |  |  |
| GA200 Laxatives | Cascara | A06AB Contact laxatives | A06AB07 Cascara |
| GA204 Stimulant laxatives | Cascara sagrada | A06AB Contact laxatives | A06AB07 Cascara |
| GA200 Laxatives | Cascara sagrada | A06AB Contact laxatives | A06AB07 Cascara |
| GA209 Laxatives,other | Cascara sagrada | A06AB Contact laxatives | A06AB07 Cascara |
| GA204 Stimulant laxatives | Cascara sagrada-phenolphth | A06AB Contact laxatives | A06AB57 Cascara, combinations |
| GA209 Laxatives,other | Cascara/magnesium hydroxide | A06AB Contact laxatives | A06AB57 Cascara, combinations |
| GA200 Laxatives | Castile soap |  |  |
| RS300 Laxatives,rectal | Castile soap |  |  |
| GA204 Stimulant laxatives | Castor oil | A06AB Contact laxatives | A06AB05 Castor oil |
| GA200 Laxatives | Castor oil | A06AB Contact laxatives | A06AB05 Castor oil |
| GA204 Stimulant laxatives | Castor oil/bisacodyl | A06AB Contact laxatives | A06AB52 Bisacodyl, combinations |
| GA201 Bulk-forming laxatives | Cellulose |  |  |
| GA202 Hyper-osmotic laxatives | Citric acid/magnesium oxide/sodium picosulfate | A06AB Contact laxatives | A06AB58 Sodium picosulfate, combinations |
| GA200 Laxatives | Danthron | A06AB Contact laxatives | A06AB03 Dantron |
| GA209 Laxatives,other | Danthron | A06AB Contact laxatives | A06AB03 Dantron |
| GA200 Laxatives | Danthron/docusate | A06AB Contact laxatives | A06AB53 Dantron, combinations |
| GA204 Stimulant laxatives | Dehydrocholic acid |  |  |
| GA200 Laxatives | Dehydrocholic acids |  |  |
| GA209 Laxatives,other | Dehydrocholic acids |  |  |
| GA203 Lubricant laxatives | Docusate | A06AG Enemas | A06AG10 Docusate sodium, incl. Combinations |
| RS300 Laxatives,rectal | Docusate | A06AG Enemas | A06AG10 Docusate sodium, incl. Combinations |
| GA200 Laxatives | Docusate |  |  |
| GA205 Stool softener | Docusate |  |  |
| GA200 Laxatives | Docusate |  |  |
| GA209 Laxatives,other | Docusate |  |  |
| GA205 Stool softener | Docusate calcium | A06AG Enemas | A06AG10 Docusate sodium, incl. Combinations |
| GA200 Laxatives | Docusate calcium |  |  |
| GA200 Laxatives | Docusate calcium/danthron | A06AB Contact laxatives | A06AB53 Dantron, combinations |
| GA200 Laxatives | Docusate calcium/phenolphthalein |  |  |
| GA209 Laxatives,other | Docusate calcium/phenolphthalein |  |  |
| GA205 Stool softener | Docusate potassium |  |  |
| GA200 Laxatives | Docusate potassium |  |  |
| GA205 Stool softener | Docusate potassium/casanthranol |  |  |
| GA209 Laxatives,other | Docusate potassium/casanthranol |  |  |
| GA205 Stool softener | Docusate sodium |  |  |
| RS300 Laxatives,rectal | Docusate sodium | A06AG Enemas | A06AG10 Docusate sodium, incl. Combinations |
| GA200 Laxatives | Docusate sodium |  |  |
| GA200 Laxatives | Docusate sodium |  |  |
| RS300 Laxatives,rectal | Docusate sodium/benzocaine | A06AG Enemas | A06AG10 Docusate sodium, incl. Combinations |
| GA200 Laxatives | Docusate sodium/benzocaine |  |  |
| GA209 Laxatives,other | Docusate sodium/benzocaine |  |  |
| GA200 Laxatives | Docusate sodium/casanthranol |  |  |
| GA209 Laxatives,other | Docusate sodium/phenolphthalein |  |  |
| GA204 Stimulant laxatives | Docusate Sodium, Psyllium Seed (With Dextrose), Bisacodyl, Phenolphthalein (Modane) |  |  |
| GA200 Laxatives | Docusate Sodium, Psyllium Seed (With Dextrose), Bisacodyl, Phenolphthalein (Modane) |  |  |
| GA200 Laxatives | Docusate/carboxymethycellulose |  |  |
| GA200 Laxatives | Docusate/casanthranol |  |  |
| GA209 Laxatives,other | Docusate/casanthranol |  |  |
| GA200 Laxatives | Docusate/danthron | A06AB Contact laxatives | A06AB53 Dantron, combinations |
| GA204 Stimulant laxatives | Docusate/danthron | A06AB Contact laxatives | A06AB53 Dantron, combinations |
| RS300 Laxatives,rectal | Docusate/glycerin | A06AG Enemas | A06AG10 Docusate sodium, incl. Combinations |
| GA200 Laxatives | Docusate/phenolphthalein |  |  |
| GA209 Laxatives,other | Docusate/phenolphthalein |  |  |
| GA200 Laxatives | Docusate/senna |  |  |
| GA204 Stimulant laxatives | Docusate/sennosides |  |  |
| GA202 Hyper-osmotic laxatives | Electrolytes/polyethylene glycol | A06AD Osmotically acting laxatives | A06AD65 Macrogol, combinations |
| GA201 Bulk-forming laxatives | Fiber |  |  |
| GA209 Laxatives,other | Fiber |  |  |
| RS300 Laxatives,rectal | Glycerin | A06AG Enemas | A06AG04 Glycerol |
| GA202 Hyper-osmotic laxatives | Glycerin | A06AX Other drugs for constipation | A06AX01 Glycerol |
| GA200 Laxatives | Glycerin | A06AX Other drugs for constipation | A06AX01 Glycerol |
| GA202 Hyper-osmotic laxatives | Glycerin/magnesium citrate |  |  |
| GA201 Bulk-forming laxatives | Guar gum |  |  |
| GA201 Bulk-forming laxatives | Inulin |  |  |
| GA201 Bulk-forming laxatives | Ispaghula husks, (Siblin) | A06AC Bulk-forming laxatives | A06AC01 ispaghula (psylla seeds); A06AC51 ispaghula, combinations |
| GA202 Hyper-osmotic laxatives | Lactulose | A06AD Osmotically acting laxatives | A06AD11 Lactulose |
| GA200 Laxatives | Lactulose | A06AD Osmotically acting laxatives | A06AD11 Lactulose |
| RS300 Laxatives,rectal | Lactulose |  |  |
| GA200 Laxatives | Lidocaine/aluminum hydroxide/magnesium hydroxide/simethicone |  |  |
| GA202 Hyper-osmotic laxatives | Magnesium citrate | A06AD Osmotically acting laxatives | A06AD19 Magnesium citrate |
| GA202 Hyper-osmotic laxatives | Magnesium citrate/bisacodyl |  |  |
| GA209 Laxatives,other | Magnesium citrate/bisacodyl |  |  |
| GA200 Laxatives | Magnesium citrate/phenolphthalein/potassium bitartrate/sodium bi |  |  |
| GA209 Laxatives,other | Magnesium citrate/phenolphthalein/potassium bitartrate/sodium bi |  |  |
| GA202 Hyper-osmotic laxatives | Magnesium citrate/polyethylene glycol/simethicone | A06AD Osmotically acting laxatives | A06AD65 Macrogol, combinations |
| GA202 Hyper-osmotic laxatives | Magnesium hydroxide |  |  |
| GA209 Laxatives,other | Magnesium hydroxide |  |  |
| GA200 Laxatives | Magnesium hydroxide/cascara |  |  |
| GA209 Laxatives,other | Magnesium hydroxide/cascara |  |  |
| GA200 Laxatives | Magnesium hydroxide/mineral oil |  |  |
| GA209 Laxatives,other | Magnesium hydroxide/mineral oil |  |  |
| GA209 Laxatives,other | Magnesium hydroxide/phenolphthalein |  |  |
| GA200 Laxatives | Magnesium oxide | A06AD Osmotically acting laxatives | A06AD02 Magnesium oxide |
| GA202 Hyper-osmotic laxatives | Magnesium sulfate | A06AD Osmotically acting laxatives | A06AD04 Magnesium sulfate |
| GA204 Stimulant laxatives | Magnesium sulfate | A06AD Osmotically acting laxatives | A06AD04 Magnesium sulfate |
| GA200 Laxatives | Magnesium sulfate | A06AD Osmotically acting laxatives | A06AD04 Magnesium sulfate |
| GA202 Hyper-osmotic laxatives | Magnesium sulfate/potassium sulfate/sodium sulfate |  |  |
| GA201 Bulk-forming laxatives | Malt |  |  |
| GA201 Bulk-forming laxatives | Methylcellulose | A06AC Bulk-forming laxatives | A06AC06 methylcellulose |
| GA203 Lubricant laxatives | Milkinol |  |  |
| GA203 Lubricant laxatives | Mineral oil |  |  |
| GA200 Laxatives | Mineral oil |  |  |
| RS300 Laxatives,rectal | Mineral oil | A06AG Enemas | A06AG06 Oil |
| GA203 Lubricant laxatives | Mineral Oil (Kondremul) |  |  |
| GA200 Laxatives | Mineral Oil (Kondremul) |  |  |
| GA200 Laxatives | Mineral Oil (Kondremul)/cascara |  |  |
| GA201 Bulk-forming laxatives | Oat bran |  |  |
| GA203 Lubricant laxatives | Petrolatum |  |  |
| GA204 Stimulant laxatives | Phenolphthalein | A06AB Contact laxatives | A06AB04 Phenolphthalein |
| GA200 Laxatives | Phenophthalein | A06AB Contact laxatives | A06AB04 Phenolphthalein |
| GA209 Laxatives,other | Phenophthalein/docusate |  |  |
| GA200 Laxatives | Polycarbophil | A06AC Bulk-forming laxatives | A06AC08 polycarbophil calcium |
| GA202 Hyper-osmotic laxatives | Polyethylene glycol | A06AD Osmotically acting laxatives | A06AD15 Macrogol |
| GA200 Laxatives | Polyethylene glycol | A06AD Osmotically acting laxatives | A06AD15 Macrogol |
| GA209 Laxatives,other | Polyethylene glycol | A06AD Osmotically acting laxatives | A06AD15 Macrogol |
| GA202 Hyper-osmotic laxatives | Polyethylene glycol/bisacodyl | A06AD Osmotically acting laxatives | A06AD65 Macrogol, combinations |
| GA202 Hyper-osmotic laxatives | Polyethylene glycol/bisacodyl/magnesium citrate | A06AD Osmotically acting laxatives | A06AD65 Macrogol, combinations |
| GA200 Laxatives | Polyethylene glycol/electrolytes | A06AD Osmotically acting laxatives | A06AD65 Macrogol, combinations |
| GA202 Hyper-osmotic laxatives | Polyethylene glycol/metoclopramide | A06AD Osmotically acting laxatives | A06AD65 Macrogol, combinations |
| GA201 Bulk-forming laxatives | Psyllium | A06AC Bulk-forming laxatives | A06AC01 ispaghula (psylla seeds); A06AC51 ispaghula, combinations |
| GA200 Laxatives | Psyllium | A06AC Bulk-forming laxatives | A06AC01 ispaghula (psylla seeds); A06AC51 ispaghula, combinations |
| RS300 Laxatives,rectal | Psyllium |  |  |
| GA200 Laxatives | Psyllium hydrophilic mucilloid | A06AC Bulk-forming laxatives | A06AC01 ispaghula (psylla seeds); A06AC51 ispaghula, combinations |
| RS300 Laxatives,rectal | Saline/mineral oil/glycerin | A06AG Enemas | A06AG20 Combinations |
| GA204 Stimulant laxatives | Senna | A06AB Contact laxatives | A06AB06 Senna glycosides |
| GA200 Laxatives | Senna | A06AB Contact laxatives | A06AB06 Senna glycosides |
| GA209 Laxatives,other | Senna | A06AB Contact laxatives | A06AB06 Senna glycosides |
| RS300 Laxatives,rectal | Senna |  |  |
| GA209 Laxatives,other | Senna/docusate | A06AB Contact laxatives | A06AB56 Senna glycosides, combinations |
| GA204 Stimulant laxatives | Sennosides | A06AB Contact laxatives | A06AB06 Senna glycosides |
| GA200 Laxatives | Sennosides | A06AB Contact laxatives | A06AB06 Senna glycosides |
| GA203 Lubricant laxatives | Sennosides A and B (Agoral) | A06AB Contact laxatives | A06AB06 Senna glycosides |
| GA200 Laxatives | Sennosides A and B (Agoral) | A06AB Contact laxatives | A06AB06 Senna glycosides |
| GA209 Laxatives,other | Sennosides A and B (Agoral) | A06AB Contact laxatives | A06AB06 Senna glycosides |
| RS300 Laxatives,rectal | Soap |  |  |
| RS300 Laxatives,rectal | Sodium bicarbonate and potassium bitartrate | A06AX Other drugs for constipation | A06AX02 Carbon dioxide producing drugs |
| GA202 Hyper-osmotic laxatives | Sodium biphosphate/sodium phosphate |  |  |
| GA202 Hyper-osmotic laxatives | Sodium phosphate | A06AD Osmotically acting laxatives | A06AD17 Sodium phosphate |
| GA200 Laxatives | Sodium phosphate | A06AD Osmotically acting laxatives | A06AD17 Sodium phosphate |
| RS300 Laxatives,rectal | Sodium phosphate | A06AG Enemas | A06AG01 Sodium phosphate |
| GA202 Hyper-osmotic laxatives | Sodium phosphate dibasic and sodium phosphate monobasic |  |  |
| GA200 Laxatives | Sodium phosphate dibasic and sodium phosphate monobasic |  |  |
| GA209 Laxatives,other | Sodium phosphate dibasic and sodium phosphate monobasic |  |  |
| RS300 Laxatives,rectal | Sodium phosphate dibasic and sodium phosphate monobasic | A06AG Enemas | A06AG20 Combinations |
| GA209 Laxatives,other | Sodium phosphate/bisacodyl | A06AB Contact laxatives | A06AB52 Bisacodyl, combinations |
| GA209 Laxatives,other | Sodium phosphate/bisacodyl/sodium phosphate dibasic and sodium phosphate monobasic | A06AB Contact laxatives | A06AB52 Bisacodyl, combinations |
| GA202 Hyper-osmotic laxatives | Sorbitol | A06AD Osmotically acting laxatives | A06AD18 Sorbitol |
| GA200 Laxatives | Sorbitol | A06AD Osmotically acting laxatives | A06AD18 Sorbitol |
| RS300 Laxatives,rectal | Sorbitol/magnesium hydroxide/mineral oil/glycerin | A06AG Enemas | A06AG07 Sorbitol |
| GA201 Bulk-forming laxatives | Wheat dextrin | A06AC Bulk-forming laxatives | A06AC07 triticum (wheat fibre) |
|  |  | A06AB Contact laxatives | A06AB01 Oxyphenisatine |
|  |  | A06AB Contact laxatives | A06AB08 Sodium picosulfate |
|  |  | A06AB Contact laxatives | A06AB09 Bisoxatin |
|  |  | A06AB Contact laxatives | A06AB20 Contact laxatives in combination |
|  |  | A06AB Contact laxatives | A06AB30 Contact laxatives in combination with belladonna alkaloids |
|  |  | A06AC Bulk-forming laxatives | A06AC02 ethulose |
|  |  | A06AC Bulk-forming laxatives | A06AC03 sterculia; A06AC53 sterculia, combinations |
|  |  | A06AC Bulk-forming laxatives | A06AC05 linseed; A06AC55 linseed, combinations |
|  |  | A06AD Osmotically acting laxatives | A06AD01 Magnesium carbonate |
|  |  | A06AD Osmotically acting laxatives | A06AD03 Magnesium peroxide |
|  |  | A06AD Osmotically acting laxatives | A06AD10 Mineral salts in combination |
|  |  | A06AD Osmotically acting laxatives | A06AD12 Lactitol |
|  |  | A06AD Osmotically acting laxatives | A06AD13 Sodium sulfate |
|  |  | A06AD Osmotically acting laxatives | A06AD14 Pentaerithrityl |
|  |  | A06AD Osmotically acting laxatives | A06AD16 Mannitol |
|  |  | A06AD Osmotically acting laxatives | A06AD21 Sodium tartrate |
|  |  | A06AD Osmotically acting laxatives | A06AD61 Lactulose, combinations |
|  |  | A06AG Enemas | A06AG03 Dantron, incl. Combinations |
|  |  | A06AG Enemas | A06AG11 Sodium lauryl sulfoacetate, incl. Combinations |
|  |  | A06AH Peripheral opioid receptor antagonists | A06AH01 Methylnaltrexone bromide |
|  |  | A06AH Peripheral opioid receptor antagonists | A06AH02 Alvimopan |
|  |  | A06AH Peripheral opioid receptor antagonists | A06AH03 Naloxegol |
|  |  | A06AH Peripheral opioid receptor antagonists | A06AH04 Naloxone |
|  |  | A06AH Peripheral opioid receptor antagonists | A06AH05 Naldemedine |
|  |  | A06AX Other drugs for constipation | A06AX03 Lubiprostone |
|  |  | A06AX Other drugs for constipation | A06AX04 Linaclotide |
|  |  | A06AX Other drugs for constipation | A06AX05 Prucalopride |
|  |  | A06AX Other drugs for constipation | A06AX06 Tegaserod |
|  |  | A06AX Other drugs for constipation | A06AX07 Plecanatide |

Notes:

Mappings to VA Drugs excludes research and non-formulary drugs (drugs only available by request).

Route was only included in the mapping if it was implied in both categories, e.g. RS300 Rectal Laxatives was only mmapped to A06AG Enemas where rectal route is consistent.

Macrogol is mapped as an index term to Polyethylene glycol in Lexicomp

Modane has been withdrawn in some countries with concern over carcinogenicity

“The Food and Drug Administration (FDA) is issuing a final rule establishing that the over-thecounter (OTC) stimulant laxative ingredients danthron and phenolphthalein are not generally recognized as safe and effective and are misbranded.(Federal Register Jan 29, 1999, p. 4535)

Dantron has also been know as danthron and was mapped as such in the comparison.

“The Food and Drug Administration (FDA) is issuing a final rule stating that the stimulant laxative ingredients aloe (including aloe extract and aloe flower extract) and cascara sagrada (including casanthranol, cascara fluidextract aromatic, cascara sagrada bark, cascara sagrada extract, and cascara sagrada fluidextract) in over-the-counter (OTC) drug products are not generally recognized as safe and effective or are misbranded.” (Federal Register May 9, 2002, p. 31125)

Table 4: CPT, ICD-9 Procedure, and HCPCS Codes used for Constipation

| **Procedure** | **CPT code** | **ICD-9 procedure code** | **HCPCS code** |
| --- | --- | --- | --- |
| Removal of impacted feces | 45915 | 96.38 |  |
| Bowel irrigation |  |  | E0350, E0352 |
| Other trans anal enema (Rectal irrigation) |  | 96.39 |  |
| Enema bag with tubing, reusable |  |  | A4458 |
| Insertion of rectal tube |  | 96.09 |  |
| Proctoclysis |  | 96.37 |  |
| Pelvic floor stimulator |  |  | E0740 |

Table 5: Cancer Diagnosis Codes for Inclusion (from ICD-9-CM 140-239 Neoplasms)

| **Codes** | **Description** |
| --- | --- |
| 140.xx – 209.3x | Malignant neoplasms of lip, oral cavity and pharynx thru malignant neoplasms of lymphatic and hematopoietic tissue |
| 230.xx – 234.xx | Carcinoma in situ thru neoplasms of unspecified nature |

Table 6: Covariates/Risk Factors

| **Variable** | **Definitions** |
| --- | --- |
| **Demographics** |  |
| Race African American | Race: African American |
| Age at Opioid Start | Calculated from Date of Birth |
| Race Other | Race: All Races other than White and African American |
| Gender, Female | Gender: Female |
| Race Unknown | Race: Blank or Unknown |
|  |  |
| **Laboratory Tests** |  |
| Albumin | Curated string search on “Albumin” and LOINC - 1746-7, 1747-5, 1748-3, 1749-1, 1751-7 |
| Bicarbonate CO2 | Curated string search on “Bicarbonate” |
| Bilirubin Total | Curated string search on “Bilirubin, Total” and LOINC - 14422-0, 14631-6, 1974-5, 1975-2, 34442-4 |
| Chloride | Curated string search on “Chloride” and LOINC - 2069-3, 2070-1, 2072-7, 2075-0, 2078-4, 2079-2, 21194-6 |
| Cholesterol HDL | Curated string search on “Cholesterol HDL” and LOINC - 17082-9, 18263-4, 2085-9, 2093-3, 2095-8, 2573-4 |
| Cholesterol LDL | Curated string search on “Cholesterol LDL” and LOINC - 12773-8, 13457-7, 17083-7, 18262-6, 2089-1 |
| Creatinine | Curated string search on “Creatinine” and LOINC - 12190-5, 12585-6, 20624-3, 2148-5, 2160-0, 2161-8, 33914-3, 34555-3, 38483-4 |
| Glucose Quant | Curated string search on “Glucose” and LOINC - 14743-9, 1558-6, 20436-2, 2339-0, 2340-8 |
| Hematocrit Macro or Micro | Curated string search on “Hematocrit” and LOINC - 11153-4, 20570-8, 32354-3, 4544-3, 4545-0 |
| Hemoglobin | Curated string search on “Hemoglobin” and LOINC - 14134-1, 14775-1, 25433-4, 30313-1, 33509-1, 717-9, 718-7, 721-1, 725-2 |
| MCH | Curated string search on “MCH” and LOINC - 28539-5, 785-6, 786-4 |
| MCHC | Curated string search on “MCHC” and LOINC - 28540-3, 785-6, 786-4 |
| MCV | Curated string search on “MCV” and LOINC - 787-2 |
| Phosphatase Alkaline | Curated string search on “Alkaline Phosphatase” and LOINC - 15148-0, 1775-6, 1777-2, 1778-0, 6768-6 |
| Platelet Estimate | Curated string search on “Platelet” and LOINC - 11125-2, 12243-2, 778-1, 9317-9 |
| Sodium | Curated string search on “Sodium” and LOINC - 13895-8, 21525-1, 2947-0, 2950-4, 2951-2, 2955-3, 2956-1, 32717-1 |
| Transferase Alanine Amino SGPT | Curated string search on “Transferase Alanine Amino” and LOINC - 1741-8, 1742-6, 1743-4, 1744-2, 25302-1 |
| Transferase Aspartate SGOT | Curated string search on “Transferase Aspartate” and LOINC - 14409-7, 1919-0, 1920-8 |
| Triglyceride | Curated string search on “Triglyceride” and LOINC - 12228-3, 2571-8 |
| Urea Nitrogen | Curated string search on “Urea Nitrogen” and LOINC - 12967-6, 17757-6, 3093-2, 3094-0, 3095-7, 3096-5, 3097-3, 5918-8, 6299-2 |
| White Blood Cell Count | Curated string search on “White Blood Cell Count” and LOINC - 11157-5, 12179-8, 13524-4, 20584-9, 26464-8, 26466-3, 6690-2, 6743-9, 804-5, 808-6, 810-2 |
|  |  |
| **Medications** |  |
| AH000, Anti-histamines | Drug class code: AH000 |
| AM200, Erythromycins/ Macrolides | Drug class code: AM200 |
| AM400, Quinolones | Drug class code: AM400 |
| AM800, Anti-virals | Drug class code: AM800 |
| BL110, Anti-coagulants | Drug class code: BL110 |
| CN309, Sedatives/Hypnotic, Other | Drug class code: CN309 |
| CN500, Anti-Parkinson Agents | Drug class code: CN500 |
| CN600, Anti-depressants | Drug class code: CN600 |
| CN700, Anti-psychotics | Drug class code: CN700 |
| CN900, CNS Medications, Other | Drug class code: CN900 |
| CV050, Digitalis Glycosides | Drug class code: CV050 |
| CV100, Beta Blockers/Related | Drug class code: CV100 |
| CV150, Alpha Blockers/Related | Drug class code: CV150 |
| CV250, Anti-anginals | Drug class code: CV250 |
| CV350, Anti-lipemic Agents | Drug class code: CV350 |
| DE650, Analgesics, Topical | Drug class code: DE650 |
| DE700, Local Anesthetics, Topical | Drug class code: DE700 |
| GA300, Anti-ulcer Agents | Drug class code: GA300 |
| GA900, Gastric Medications, Other | Drug class code: GA900 |
| GU000, Genitourinary Medications | Drug class code: GU000 |
| HS050, Adrenal Corticosteriods | Drug class code: HS050 |
| HS100, Androgens Anabolics | Drug class code: HS100 |
| HS501, Insulin | Drug class code: HS501 |
| HS502, Oral Hypoglycemic Agents, Oral | Drug class code: HS502 |
| HS850, Thyroid Modifiers | Drug class code: HS850 |
| HS900, Hormones/Synthetic/Modifiers, Other | Drug class code: HS900 |
| MS102, Nonsalicylate NSAIs, Anti-rheumatic | Drug class code: MS102 |
| MS400, Anti-gout Agents | Drug class code: MS400 |
| TN430, Potassium | Drug class code: TN430 |
| VT000, Vitamins | Drug class code: VT000 |
| Laxatives Any Route Not Prevalent | See Table 3 |
| Prevalent Laxative | See Table 3 |
| Preventative Laxative, and const. within a year | See Table 2, 3, and 4 |
| Preventative Laxative, and no const. within a year | See Table 2, 3, and 4 |
| Prior Use 1 Year To 61 Days | Constipating medications were handled as a special class and developed using the definition of anti-cholinergic drugs identified by Boustani and colleagues, and supplemented by a group of pharmacy industry experts (see Table 8). |
| Prior Use 30 to 1 Days |  |
| Prior Use 60 to 31 Days |  |
|  |  |
| **Administrative Code Based Conditions** |  |
| Cerebrovascular Disease | \| Any 2 outpatient diagnoses OR 1 inpatient diagnosis found \| Code Type Value  ICD 43[0-2]%  ICD 433.[0-389]1  ICD 434.[019]  ICD 434.[019]1  ICD 435.[89]  ICD 43[6-8]%  ICD 997.02 \| \| --- \| --- \| |
| Chronic Kidney Disease | \| Any 2 outpatient diagnoses OR 1 inpatient diagnosis found \| Code Type Value  ICD 585%  ICD 403%  ICD 404% \| \| --- \| --- \| |
| Congestive Heart Failure | \| Deyo's ICD-9-CM Charlson Comorbidity definition from Quan Med Care 2005;43:1130-1139 \| Any 2 outpatient diagnoses OR 1 inpatient diagnosis found \| Code Type Value  ICD 428% \| \| --- \| --- \| --- \| |
| Constipation Within a Year | See Appendix 1, Table 2-4 |
| Coronary Artery Disease | \| Solberg PMID: 16849780 with V codes added \| Any 2 outpatient diagnoses OR 1 inpatient diagnosis found \| Code Type Value  ICD 410.%  ICD 411.%  ICD 412.%  ICD 413.%  ICD 414.%  ICD V45.81  ICD V45.82 \| \| --- \| --- \| --- \| |
| Diabetes Mellitus | \| Does not include pregnancy related codes \| Any 2 outpatient diagnoses OR 1 inpatient diagnosis found \| Code Type Value  ICD 249%  ICD 250%  ICD 357.2%  ICD 362.0%  ICD 366.41  ICD V45.85  ICD V53.91 \| \| --- \| --- \| --- \| |
| Diabetic Neuropathy | \| Any 2 outpatient diagnoses OR 1 inpatient diagnosis found \| Code Type Value  ICD 357.2 \| \| --- \| --- \| |
| Fibromyalgia | \| Any 2 outpatient diagnoses OR 1 inpatient diagnosis found \| Code Type Value  ICD 729.1 \| \| --- \| --- \| |
| GI Anatomical Trauma | Includes gastrointestinal perforation (ICD 569.83, bowel perforation (ICD 569.83), rectal prolapse (ICD 569.1), rectocele (ICD 618.04), megacolon (ICD 564.7), impaction (ICD 560.32), and anal fissures fistula (ICD 565.1) |
| Hemorrhoids | \| Any 1 \| Code Type Value  ICD 455.[0-9] \| \| --- \| --- \| |
| Hyperkalemia | \| Any 2 outpatient diagnoses OR 1 inpatient diagnosis found \| Code Type Value  ICD 276.7 \| \| --- \| --- \| |
| Hypertension | \| Does not include pregnancy related codes \| Any 2 outpatient diagnoses OR 1 inpatient diagnosis found \| Code Type Value  ICD 401%  ICD 402%  ICD 403%  ICD 404%  ICD 405%  ICD 437.2% \| \| --- \| --- \| --- \| |
| Irritable Bowel Syndrome | \| Any 2 outpatient diagnoses OR 1 inpatient diagnosis found \| Code Type Value  ICD 564.1 \| \| --- \| --- \| |
| Migraine Headache | \| Any 2 outpatient diagnoses OR 1 inpatient diagnosis found \| Code Type Value  ICD 784.0  ICD 339.0[0123459]  ICD 339.[12][0-2]  ICD 339.3  ICD 339.4[1-4]  ICD 339.8[123459]  ICD 346.[0-5][0-3]  ICD 346.[0-2]  ICD 346.[7-9][0-3]  ICD 346.[89] \| \| --- \| --- \| |
| Myocardial Infarction | \| Any 2 outpatient diagnoses OR 1 inpatient diagnosis found \| Code Type Value  ICD 410% \| \| --- \| --- \| |
| Myopathies | \| Any 2 outpatient diagnoses OR 1 inpatient diagnosis found \| Code Type Value  ICD 359.8  ICD 359.89  ICD 425.4 \| \| --- \| --- \| |
| Osteoarthritis | \| Any 1 \| Code Type Value  ICD 715.0[049]  ICD 715.1[0-8]  ICD 715.2[0-8]  ICD 715.3[0-8]  ICD 715.8[09]  ICD 715.9[0-8]  ICD V13.4 \| \| --- \| --- \| |
| Revascularization CABG | \| 2 Outpt Dx, 1 Outpt Pr, 1 Inpt Dx, or 1 Inpt Proc \| Code Type Value  CPT 3351[012346789]  CPT 3352[123]  CPT 3353[3-6]  ICD V45.81  ICD 414.04  ICDProc 36.1%  ICDProc 36.2% \| \| --- \| --- \| |
| Rheumatoid Arthritis | \| Any 2 outpatient diagnoses OR 1 inpatient diagnosis found \| Code Type Value  ICD 714.0 \| \| --- \| --- \| |
| Stable Angina | \| Any 1 \| Code Type Value  ICD 413.[019] \| \| --- \| --- \| |
| Stroke | \| Any 2 outpatient diagnoses OR 1 inpatient diagnosis found \| Code Type Value  ICD 43[01]%  ICD 434.[019]  ICD 434.[019]1  ICD 436%  ICD 997.02 \| \| --- \| --- \| |
| Unstable Angina | \| Any 1 \| Code Type Value  ICD 411.1%  ICD 413% \| \| --- \| --- \| |

The ‘%’ symbol is an unconditional wild card not limited to number or type of characters. Code types of ICD are limited to ICD9 as the study dates are all prior to the implementation of ICD10. Brackets ‘[]’ describe single character/number values that would be considered valid.

Table 7: Excluded Laboratory Tests

| **Variable** |
| --- |
| Ammonia |
| Sodium Urine |
| Creatine Kinase (CK MB) |
| Transpeptidase Gamma Glut |
| Natriuretic Peptide Brain (B Type) |
| Cholesterol VLDL |
| Creatine Kinase (CK) |
| Thromboplastin Time Partial |
| Bilirubin Direct |
| Prothrombin Time |
| Protein Stick |

Table 8: Constipating Medication

| **Drug Name** |
| --- |
| Acebutolol |
| Acetaminophen/chlorpheniramine |
| Acetaminophen/chlorpheniramine/dextromethorphan |
| Acetaminophen/chlorpheniramine/dextromethorphan/pseudoephedrine |
| Acetaminophen/chlorpheniramine/phenylephrine |
| Acetaminophen/chlorpheniramine/pseudoephedrine |
| Acetaminophen/diphenhydramine |
| Acetaminophen/diphenhydramine/phenylephrine |
| Acetaminophen/pseudoephedrine |
| Alendronate |
| Alendronate/cholecalciferol |
| Aliskiren |
| Aliskiren/amlodipine |
| Aliskiren/amlodipine/hydrochlorothiazide |
| Aliskiren/hydrochlorothiazide |
| Aliskiren/valsartan |
| Alprazolam |
| Amiloride |
| Amiloride/hydrochlorothiazide |
| Amitriptyline |
| Amitriptyline/chlordiazepoxide |
| Amitriptyline/perphenazine |
| Amlodipine |
| Amlodipine/atorvastatin |
| Amlodipine/benazepril |
| Amlodipine/hydrochlorothiazide/olmesartan |
| Amlodipine/hydrochlorothiazide/valsartan |
| Amlodipine/olmesartan |
| Amlodipine/telmisartan |
| Amlodipine/valsartan |
| Amoxapine |
| Amphetamine resin complex |
| Amphetamine/dextroamphetamine |
| Anastrozole |
| Aprepitant |
| Armodafinil |
| Arsenic |
| Ascorbic acid |
| Ascorbic acid/cyanocobalamin/ferrous fumarate |
| Ascorbic acid/ferrous sulfate/folic acid |
| Ascorbic acid/iron |
| Aspirin |
| Aspirin/caffeine/orphenadrine |
| Aspirin/carisoprodol |
| Aspirin/chlorpheniramine/dextromethorphan/phenylephrine |
| Aspirin/chlorpheniramine/phenylephrine |
| Atenolol |
| Atenolol/chlorthalidone |
| Atropine |
| Atropine/benzoic/hyoscyamine/methenamine/methylene/phenyl |
| Atropine/difenoxin |
| Atropine/diphenoxylate |
| Atropine/edrophonium |
| Atropine/hyoscyamine/phenobarbital/scopolamine |
| Atropine/pralidoxime |
| Azilsartan |
| Azilsartan/chlorthalidone |
| Baclofen |
| Basiliximab |
| Benazepril/hydrochlorothiazide |
| Bendamustine |
| Bendroflumethiazide/nadolol |
| Benztropine |
| Betaxolol |
| Bicalutamide |
| Bisoprolol |
| Bisoprolol/hydrochlorothiazide |
| Bortezomib |
| Brompheniramine |
| Brompheniramine/dextromethorphan/phenylephrine |
| Brompheniramine/dextromethorphan/pseudoephedrine |
| Brompheniramine/pseudoephedrine |
| Bupropion |
| Busulfan |
| Butabarbital/hyoscyamine/phenazopyridine |
| Calcium |
| Calcium acetate |
| Calcium carbonate |
| Calcium carbonate/famotidine/magnesium hydroxide |
| Calcium carbonate/folic acid/magnesium carbonate |
| Calcium carbonate/magnesium carbonate |
| Calcium carbonate/magnesium hydroxide |
| Calcium chloride |
| Calcium citrate |
| Calcium glubionate |
| Calcium gluconate |
| Calcium glycerophosphate |
| Calcium lactate |
| Candesartan/hydrochlorothiazide |
| Captopril |
| Captopril/hydrochlorothiazide |
| Carbamazepine |
| Carbetapentane/chlorpheniramine/ephedrine/phenylephrine |
| Carbinoxamine |
| Carboplatin |
| Carmustine |
| Chlordiazepoxide |
| Chlordiazepoxide/clidinium |
| Chlorothiazide |
| Chlorpheniramine |
| Chlorpheniramine/chlophedianol/pseudoephedrine |
| Chlorpheniramine/codeine/pseudoephedrine |
| Chlorpheniramine/dextromethorphan |
| Chlorpheniramine/dextromethorphan/phenylephrine |
| Chlorpheniramine/dextromethorphan/pseudoephedrine |
| Chlorpheniramine/hydrocodone |
| Chlorpheniramine/ibuprofen/pseudoephedrine |
| Chlorpheniramine/methscopolamine |
| Chlorpheniramine/methscopolamine/phenylephrine |
| Chlorpheniramine/phenylephrine |
| Chlorpheniramine/pseudoephedrine |
| Chlorpromazine |
| Chlorthalidone |
| Chlorthalidone/clonidine |
| Cholestyramine |
| Cimetidine |
| Clemastine |
| Clobazam |
| Clomipramine |
| Clonazepam |
| Clonidine |
| Clorazepate |
| Clozapine (clozaril) |
| Clozapine (fazaclo) |
| Clozapine (mylan) |
| Clozapine (teva) |
| Clozapine (udl) |
| Clozapine (versacloz) |
| Codeine/phenylephrine/promethazine |
| Codeine/promethazine |
| Colesevelam |
| Colestipol |
| Cyclobenzaprine |
| Cyclosporine |
| Dantrolene |
| Darifenacin |
| Desipramine |
| Dexamethasone |
| Dexbrompheniramine/pseudoephedrine |
| Dexchlorpheniramine |
| Dextroamphetamine |
| Dextromethorphan/promethazine |
| Dextrose/levulose/phosphoric acid |
| Diazepam |
| Dicyclomine |
| Diethylpropion |
| Diflunisal |
| Diltiazem |
| Dimenhydrinate |
| Diphenhydramine |
| Diphenhydramine/ibuprofen |
| Diphenhydramine/lidocaine/nystatin |
| Divalproex |
| Docetaxel |
| Docusate/ferrous fumarate |
| Dolasetron |
| Doxepin |
| Doxylamine/pyridoxine |
| Dronabinol |
| Edrophonium |
| Enalapril |
| Enalapril/hydrochlorothiazide |
| Enalaprilat |
| Eplerenone |
| Eprosartan |
| Eprosartan/hydrochlorothiazide |
| Eslicarbazepine |
| Esmolol |
| Esomeprazole |
| Esomeprazole/naproxen |
| Estazolam |
| Ethinyl estradiol/ferrous fumarate/norethindrone |
| Ethosuximide |
| Ethotoin |
| Etodolac |
| Everolimus |
| Ezetimibe |
| Ezetimibe/simvastatin |
| Ezogabine |
| Famotidine |
| Famotidine/ibuprofen |
| Felbamate |
| Felodipine |
| Fenoldopam |
| Fenoprofen |
| Ferric carboxymaltose |
| Ferrous fumarate |
| Ferrous fumarate/iron |
| Ferrous gluconate |
| Ferrous sulfate |
| Ferumoxytol |
| Flavoxate |
| Fluoxetine/olanzapine |
| Flurazepam |
| Flurbiprofen |
| Folic acid/iron/multivitamins |
| Fosaprepitant |
| Fosinopril |
| Fosinopril/hydrochlorothiazide |
| Fosphenytoin |
| Fulvestrant |
| Furosemide |
| Gabapentin |
| Gabapentin enacarbil |
| Granisetron |
| Guanfacine |
| Homatropine |
| Homatropine/hydrocodone |
| Hydralazine |
| Hydralazine/isosorbide |
| Hydrochlorothiazide |
| Hydrochlorothiazide/irbesartan |
| Hydrochlorothiazide/lisinopril |
| Hydrochlorothiazide/losartan |
| Hydrochlorothiazide/methyldopa |
| Hydrochlorothiazide/metoprolol |
| Hydrochlorothiazide/moexipril |
| Hydrochlorothiazide/olmesartan |
| Hydrochlorothiazide/propranolol |
| Hydrochlorothiazide/quinapril |
| Hydrochlorothiazide/spironolactone |
| Hydrochlorothiazide/telmisartan |
| Hydrochlorothiazide/triamterene |
| Hydrochlorothiazide/valsartan |
| Hydrocodone/ibuprofen |
| Hydroxyamphetamine/tropicamide |
| Hydroxyzine |
| Hyoscyamine |
| Hyoscyamine/methenamine/methylene blue/phenyl salicylate/sodium |
| Hyoscyamine/methenamine/methylene/phenyl salicyl/sodium phos |
| Ibandronate |
| Ibuprofen |
| Ibuprofen/phenylephrine |
| Ibuprofen/pseudoephedrine |
| Ifosfamide |
| Ifosfamide/mesna |
| Imipramine |
| Indapamide |
| Indomethacin |
| Infliximab |
| Irbesartan |
| Iron |
| Isradipine |
| Ketoprofen |
| Ketorolac tromethamine |
| Lacosamide |
| Lamotrigine |
| Letrozole |
| Levetiracetam |
| Lidocaine |
| Lisdexamfetamine |
| Lisinopril |
| Lorazepam |
| Losartan |
| Lovastatin |
| Lovastatin/niacin |
| Meclizine |
| Meclofenamate |
| Mefenamic acid |
| Meloxicam |
| Meperidine |
| Methamphetamine |
| Methenamine/na biphospha/phenyl salicylate/methelene/hyoscyamine |
| Methocarbamol |
| Methohexital |
| Methscopolamine |
| Methscopolamine/pseudoephedrine |
| Methsuximide |
| Methyclothiazide |
| Methyldopa |
| Methyldopate |
| Methylphenidate |
| Metoclopramide |
| Metoprolol |
| Midazolam |
| Minerals/multivitamins |
| Modafinil |
| Mycophenolate mofetil |
| Mycophenolic acid |
| Nabilone |
| Nabumetone |
| Nadolol |
| Naproxen |
| Naproxen/pseudoephedrine |
| Naproxen/sumatriptan |
| Nebivolol |
| Netupitant/palonosetron |
| Niacin/simvastatin |
| Nicardipine |
| Nifedipine |
| Nimodipine |
| Nisoldipine |
| Nortriptyline |
| Olanzapine |
| Olmesartan |
| Omeprazole |
| Omeprazole/sodium bicarbonate |
| Ondansetron |
| Orphenadrine citrate |
| Oxaliplatin |
| Oxaprozin |
| Oxazepam |
| Oxcarbazepine |
| Oxybutynin chloride |
| Oxycodone/ibuprofen |
| Paclitaxel |
| Palonosetron |
| Pamidronate |
| Paroxetine |
| Penbutolol |
| Perampanel |
| Perindopril |
| Perphenazine |
| Phenobarbital |
| Phentermine |
| Phentermine/topiramate |
| Phenylephrine |
| Phenylephrine/promethazine |
| Phenytoin |
| Piroxicam |
| Pitavastatin |
| Prazosin |
| Pregabalin |
| Primidone |
| Procarbazine |
| Prochlorperazine |
| Promethazine |
| Propantheline |
| Propranolol |
| Quazepam |
| Quetiapine |
| Quinapril |
| Ramipril |
| Risedronate |
| Rituximab |
| Rosuvastatin |
| Rufinamide |
| Scopolamine |
| Simvastatin |
| Simvastatin/sitagliptin |
| Sirolimus |
| Spironolactone |
| Sulindac |
| Tacrolimus |
| Telmisartan |
| Temazepam |
| Temozolomide |
| Terazosin |
| Thioridazine |
| Tiagabine |
| Tiludronate |
| Tolmetin |
| Tolterodine |
| Topiramate |
| Torsemide |
| Trandolapril |
| Trandolapril/verapamil |
| Tretinoin |
| Triazolam |
| Trifluoperazine |
| Trihexyphenidyl |
| Trimethobenzamide |
| Trimipramine |
| Valproate sodium |
| Valproic acid |
| Valsartan |
| Verapamil |
| Vigabatrin |
| Vinblastine |
| Vincristine |
| Vinorelbine |
| Ziconotide |
| Zoledronic |
| Zonisamide |

Table 9: Patient Characteristics Used for Imputation of Missing Data

| **Patient Characteristic** |
| --- |
| Age at Opioid Start |
| Time to Event |
| Outcome Event |
| Gender |
| Constipation Within One Year |
| Prior Use 1 Year To 61 Days |
| Prior Use 60 to 31 Days |
| Prior Use 30 to 1 Days |
| Cerebrovascular Disease |
| Chronic Kidney Disease |
| Hypertension |
| Osteoarthritis |
| Revascularization CABG |
| Rheumatoid Arthritis |
| Stable Angina |
| Stroke |
| Unstable Angina |
| Race Unknown |
| Race African American |
| Race Asian |
| Race Indian American Alaskan Native |

Table 10: Laboratory Tests Used for Imputation of Missing Data

| **Laboratory Test** |
| --- |
| Albumin |
| Bicarbonate CO2 |
| Bilirubin Total |
| Chloride |
| Cholesterol HDL |
| Cholesterol LDL |
| Creatinine |
| Glucose Quant |
| Hematocrit Macro or Micro |
| Hemoglobin |
| MCH |
| MCHC |
| MCV |
| Phosphatase Alkaline |
| Platelet Estimate |
| Sodium |
| Transferase Alanine Amino SGPT |
| Transferase Aspartate SGOT |
| Triglyceride |
| Urea Nitrogen |
| White Blood Cell Count |
